# No Strategy Can Win in the Repeated Prisoner's Dilemma: Linking Game Theory and Computer Simulations

**DOI:** 10.3389/frobt.2018.00102

**Published:** 2018-08-29

**Authors:** Julián García, Matthijs van Veelen

**Affiliations:** ^1^Faculty of Information Technology, Monash University, Melbourne, VIC, Australia; ^2^Department of Economics, Universiteit van Amsterdam, Amsterdam, Netherlands

**Keywords:** evolution, game theory, simulations, cooperation, evolutionary computation

## Abstract

Computer simulations are regularly used for studying the evolution of strategies in repeated games. These simulations rarely pay attention to game theoretical results that can illuminate the data analysis or the questions being asked. Results from evolutionary game theory imply that for every Nash equilibrium, there are sequences of mutants that would destabilize them. If strategies are not limited to a finite set, populations move between a variety of Nash equilibria with different levels of cooperation. This instability is inescapable, regardless of how strategies are represented. We present algorithms that show that simulations do agree with the theory. This implies that cognition itself may only have limited impact on the cycling dynamics. We argue that the role of mutations or exploration is more important in determining levels of cooperation.

## 1. Introduction

Costly cooperation—where individuals reduce their own fitness in order to increase somebody else's—is ubiquitous in the natural world. It is however not immediately clear why costly cooperation would have survived a process of mutation and selection. A parallel problem arises in engineering, when autonomous agents need to learn how to cooperate with each other. If agents respond to individual rewards, there is little incentive for groups of agents to behave cooperatively (Shoham and Leyton-Brown, 2008). Recognized by Darwin himself (Darwin, 1859), the problem of cooperation is still considered one of the biggest open problems in science (Pennisi, 2005).

Cooperation problems arise due to a mismatch between individual incentives and collective goals. This tension is best captured by the prisoner's dilemma (Rapoport and Chammah, 1965). Assuming that players have two possible actions, cooperate and defect, the game is specified by payoffs *R*, *S*, *T*, and *P*, such that *T*>*R*>*P*>*S*. The payoff for mutual cooperation is *R*; the payoff for mutual defection is *P*; *S* is the payoff for a cooperator that meets a defector; and a defector meeting a cooperator will get *T*. Regardless of what the other player does, the payoff of playing defect is larger than the payoff of playing cooperate, while the payoff of mutual cooperation is higher than the payoff of mutual defection. Thus, rational players will end up in the less desirable outcome of mutual defection.

Explaining the emergence of cooperation requires a mechanism that keeps the cooperators from losing ground to defectors. Such a mechanism will have to offset the costs of cooperation by causing cooperators to also be on the receiving end more often. One set of mechanisms is population structure in general, which causes deviations from random matching, and includes networks, group selection, and other spatial structures (Nowak, 2006b). Such deviations from random matching can induce kin selection. Another mechanism is indirect reciprocity based on reputation (Axelrod and Hamilton, 1981). Cooperation can be sustained if players are uncertain about when the game ends, and the probability of repetition is large enough. While repeated interactions can sustain strategies that behave cooperatively, strategies that do not cooperate can also be stable (Fudenberg and Maskin, 1986).

To see how direct reciprocity can sustain cooperation, consider a prisoner's dilemma that is repeated for an uncertain, but, in expectation, large number of rounds. A strategy that always cooperates (ALLC) will be invaded by a strategy that always defects (ALLD), because the latter always exploits the former. The strategy “Tit-for-Tat” (TFT) cooperates on the first round and repeats the previous move of its opponent thereafter. TFT against itself results in mutual cooperation. ALLD will exploit TFT in the first round, but will receive the payoff of mutual defection thereafter. Because the game in expectation is repeated many times, the advantage of exploiting the other in the first round is more than offset by the disadvantage of receiving the payoff of mutual defection instead of the payoff of mutual cooperation in subsequent rounds. Thus, players using TFT will have little incentive to switch to ALLD, and a mutually cooperative outcome becomes feasible.

With different possibilities for equilibrium behavior—both TFT and ALLD for instance are equilibria—one question could be if there is an overall best way to play a repeated prisoner's dilemma. In a pioneering study, Axelrod (1984) requested programmable strategies from game theorists, and pitted them against each other in a round-robin computer tournament, where each game was repeated two hundred times. The strategy TFT, described above, was the winner of that tournament. Axelrod's pioneering research was followed by a wealth of interdisciplinary research, cutting across fields and techniques such as evolutionary dynamics, game theory, and computer science. After TFT won Axelrod's tournament, some other studies have also declared other winners; for instance Nowak and Sigmund (1993) suggested that win-stay, lose-shift (WSLS) was a better strategy. Both simulations and game theoretical analyses are commonly used.

Recent game theoretical work shows that no equilibrium is fundamentally more stable than any other equilibrium. In this paper we will show that this prediction should also hold for studies based on simulations, provided the data are analyzed properly. This finding has strong implications for studies of cognition and cooperation. Given that game theoretical results predict instability without imposing any restrictions on the strategies, no matter how cognitively sophisticated, the instability of cooperation is inescapable. We argue that the role of mutations, or how agents explore the strategy space is more important in predicting whether cooperation is more or less likely.

The rest of the paper is organized as follows. Section 2 describes how different techniques have approached the problem of cooperation in repeated games. Our goal is to show that these different approaches are not only compatible, but also powerful when used together. Section 3 summarizes recent game theoretical findings, and delineates what properties should be present in a meaningful simulation program. One such simulation program is presented in section 4. Our main contributions are presented in section 5, where we formulate algorithms to analyse the data from the simulations. These include an algorithm to capture transitions between prevalent strategies in a noisy simulation, as well as a way to determine if prevalent strategies are Nash equilibria, as predicted by the theory. Finally section 6 concludes by discussing the implications of our results.

## 2. An overview of different approaches

### 2.1. Evolutionary game theory

In evolutionary game theory the focus of models is naturally on evolution, typically assuming that there is a population of strategies, competing for a place in the next generation based on the payoffs they get from interacting with others (Nowak, 2006a). The length of the game is uncertain, and the probabilities of different lengths are implied by the continuation probability δ, which makes the probability of *k* repetitions follow a geometric distribution with success probability δ. Sometimes the length of the game is described by the expected number of rounds. If δ is the continuation probability, then $\frac{1}{1-\delta }$ is the expected number of rounds.

In this literature, the set of strategies is often restricted for mathematical tractability. Many studies consider what is known as reactive strategies (Nowak and Sigmund, 1992). These are triplets (*y, p, q*), where *y* is the probability of cooperating in the first round, *p* is the probability of cooperating if the opponent cooperated on the last round, and *q* is the probability of cooperating if the opponent defected in the last round. Three of the strategies discussed in section 1 are included in this strategy space; TFT is represented by (1, 1, 0); ALLC is (1, 1, 1); and ALLD is (0, 0, 0).

In this approach the relative simplicity of strategies often allows for a full description of the dynamics. For example, Nowak and Sigmund (1990) derive exact equations for the evolutionary dynamics in this set, when the population is large and homogeneous. A homogeneous population hampers the chances of TFT to establish cooperation, but using computer simulations it has been shown that TFT can lead the way for cooperation when there is heterogeneity (Nowak and Sigmund, 1992).

With reactive strategies, behavior depends only on what the opponent did in the last round. Alternatively, one could also make behavior depend, not just on what the other did, but also on what one did oneself in the previous round. Combining the two would amount to four possible combinations of moves from the previous round. A vector (*p*_0_, *p*_*CC*_, *p*_*CD*_, *p*_*DC*_, *p*_*DD*_) encodes a strategy that cooperates with probability *p*_0_ in the first round, and with the other four probabilities, depending on the four possible combinations of actions in the previous round [see for instance, Hilbe et al. (2015)]. Such strategies are typically referred to as memory-1 strategies. The set of reactive strategies is a proper subset of the set of memory-1 strategies; any reactive strategy (*y, p, q*) can be rewritten as a memory-1 strategy; just choose (*y, p, q, p, q*). The strategy WSLS however is a memory-1 strategy, but not a reactive one. This strategy is represented by (1, 1, 0, 0, 1), and it repeats its own move from the previous round if the last round yielded *T* or *R*, and switches actions if the payoff was *P* or *S*.

Cyclical dynamics are a typical finding. WSLS for instance outperforms TFT if players occasionally make mistakes, but WSLS itself can still be invaded by unconditional defection. The dynamics will take a population from highly cooperative states, based on TFT-like or WSLS-like strategies, to defection and back. These cycles are a fundamental feature, already present in Axelrod (1987b). Cycles also appear when strategies are deterministic, as in for example in Imhof et al. (2005) or van Veelen et al. (2012).

Recent research has revealed that a certain kind of memory-1 strategies can be successful in the sense that they can always enforce a linear relationship between payoffs, only under the control of one player Press and Dyson (2012). Such “extortion” strategies can for instance guarantee that a player will always get twice the payoff of her opponent. In an evolutionary competition, these strategies can be catalysts of cooperation, but are never a stable outcome of evolution (Hilbe et al., 2013).

### 2.2. Notions of equilibria in games of direct reciprocity

The standard approach from game theory is to ask under what conditions a strategy will be stable, in the sense that a rational player would not have an incentive to switch to any other strategy. Absent any incentive to deviate, such a strategy is then a Nash equilibrium. What is and what is not an equilibrium of a repeated game will depend on which strategies for playing the repeated game are allowed for. In many papers, all mappings from the set of all possible possible histories (including the empty history) into an action, *C* or *D*, are included. For the repeated prisoner's dilemma, the relevant equilibrium concepts, besides the Nash equilibrium (NE), are the Evolutionarily Stable Strategy (ESS), the Neutrally Stable Strategy (NSS), and Robustness Against indirect Invasions (RAII).

The Nash equilibrium Nash (1950) is the prime solution concept in game theory. A Nash equilibrium arises when all players choose a strategy such that no unilateral deviations are profitable. In a repeated prisoner's dilemma in which the game is repeated a finite and known number of times, the only subgame perfect Nash equilibrium is for everybody to play ALLD (Cressman, 1996). To see this, it suffices to realize that the last round carries no possibility of retaliation, and thus rational players will defect. With unconditional defection in the last round, defection in the second-to-last round must become the rational thing to do too, and repeating this argument implies that playing *D* in all rounds is the only possibility for equilibrium behavior.

For the game to allow for cooperative behavior in equilibrium, the number of rounds must be uncertain. We can achieve this by assuming that each shot is repeated with continuation probability δ. This is also known as an infinitely repeated game with discounting. In the repeated prisoner's dilemma (with discounting) there is an infinite number of Nash equilibria. This follows from the Folk theorem, which asserts that for large enough δ, all payoff pairs in which both players get at least the mutual defection payoff can arise in equilibrium (Fudenberg and Maskin, 1986). This means that we can expect cooperative as well as uncooperative outcomes.

**Figure 1:**
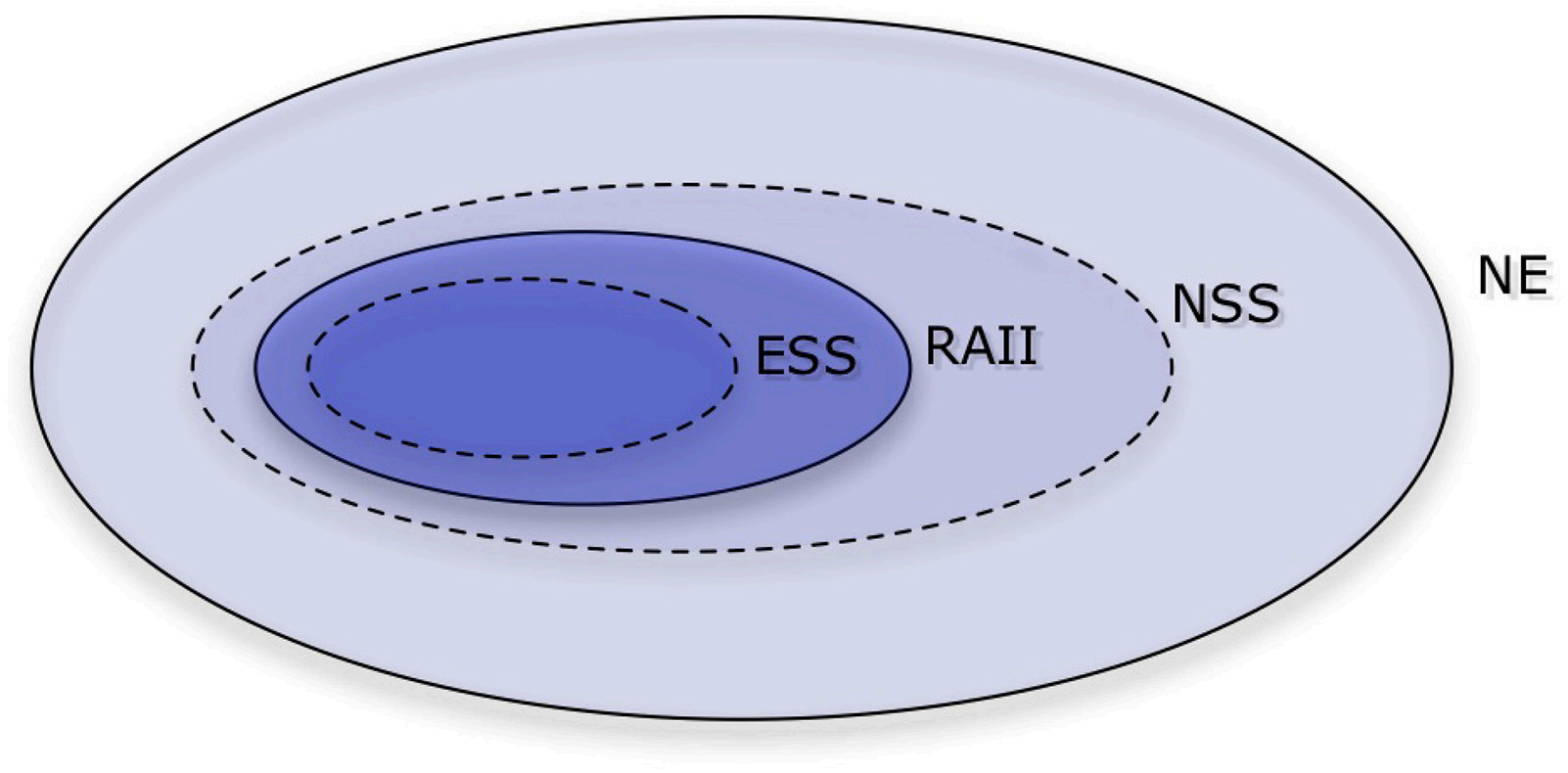
A Venn diagram of the Nash equilibrium and its refinements. Those refinements are Evolutionarily stable strategy (ESS), Robust against indirect invasions (RAII), and Neutrally stable strategy (NSS), with ESS being the tightest. In the repeated prisoner's dilemma with sufficiently many repetitions, there are no ESS'es nor strategies that are RAII, and there are infinitely many Nash equilibria and NSS'es.

The multiplicity of Nash equilibria precludes a straightforward prediction and demands a so-called *refinement*, in which the conditions on the equilibria are tightened—see Figure 1. The concept of an ESS is a natural refinement here (Maynard Smith and Price, 1973). This concept envisions an infinite population of strategies in evolutionary competition, in which payoffs (or fitness values) are determined by averaging random encounters in the population. A strategy is ESS if it outperforms any mutant, as long as the mutant arises in small enough proportions. All ESS'es are Nash equilibria, but not all Nash equilibria are ESS'es. Unfortunately, in the repeated prisoner's dilemma there is no strategy that is ESS Selten and Hammerstein (1984).

Because this solution concept leaves us with no strategies, it is reasonable to try to use a concept that is more restrictive than the Nash equilibrium, but less so than ESS. A NSS is a strategy that is able to perform at least as good as (but not necessarily strictly better than) any mutant arising in small enough fractions (Maynard Smith, 1982). Unfortunately, there are also infinitely many NSS'es in the repeated prisoner's dilemma (Bendor and Swistak, 1995).

Not all NSS are equally stable though. This may seem counter-intuitive, because by definition they cannot be invaded, except that strategies that perform just as well, also known as neutral mutants, can drift into the population. What is possible, however, is that some NSS'es will allow for mutants that open the door to other strategies that could not invade by themselves. These stepping stone paths are called indirect invasions (van Veelen, 2012). For the sake of illustration, consider the strategies TFT, ALLC, and ALLD. While TFT is a Nash equilibrium—provided the continuation probability is large enough—it is not an ESS, since ALLC will perform just as well against TFT as TFT does against itself. Moreover, an indirect invasion is possible. In a population composed of both TFT and ALLC, if ALLC is sufficiently abundant, a mutant ALLD can invade the population by exploiting ALLC. Thus, ALLD cannot invade TFT alone, but it can indirectly invade with the help of neutral mutant ALLC.

An NSS that can resist indirect invasions is called robust against indirect invasions (RAII, van Veelen (2012)). Unfortunately, there is no strategy that is RAII in the repeated prisoner's dilemma. Given any Nash equilibria, it is possible to build a path out, in which a neutral mutant opens the door to a different strategy that would not have been able to invade on its own Garćıa and van Veelen (2016). What this means is that we should expect cycles of cooperation and defection. This is in line with some of the previous studies of evolutionary dynamics. We will discuss these findings in more detail in section 3.

Altogether, the game theory literature on repeated games is enormous. Some of the richness has also carried over to the literature on evolution in repeated games. In order to be able to measure complexity, Rubinstein (1986) and Abreu and Rubinstein (1988) limited attention to finite state automata. Binmore and Samuelson (1992) and Volij (2002) did the same in order to be able to define an evolutionary stability concept that also accounts for complexity [see also Cooper (1996); Samuelson and Swinkels (2003); van Veelen and García (2012)]. Evolution of strategies with vanishing error rates is considered by Fudenberg and Maskin (1990).

### 2.3. Typical implementations

Computer scientist have been interested in the repeated prisoner's dilemma since Axelrod's famous computer tournament. They have particularly worked on the computational aspects of implementing strategies. Roughly speaking, there are two traditional approaches with roots in computer science. Computational theorists have used computational complexity theory as a way to study the algorithmic demands of implementing certain strategies; i.e., the computational complexity of a strategy is a way to formalize boundedly rational agents (Papadimitriou and Yannakakis, 1994). On the other hand, those interested in multi-agent and complex systems have tried to understand how groups of agents can learn, by following simple rules, to play the game. The latter mostly rely on the simulation of an evolutionary process, in which a particular implementation is assumed for the strategies (Axelrod, 1997).

**Figure 2:**
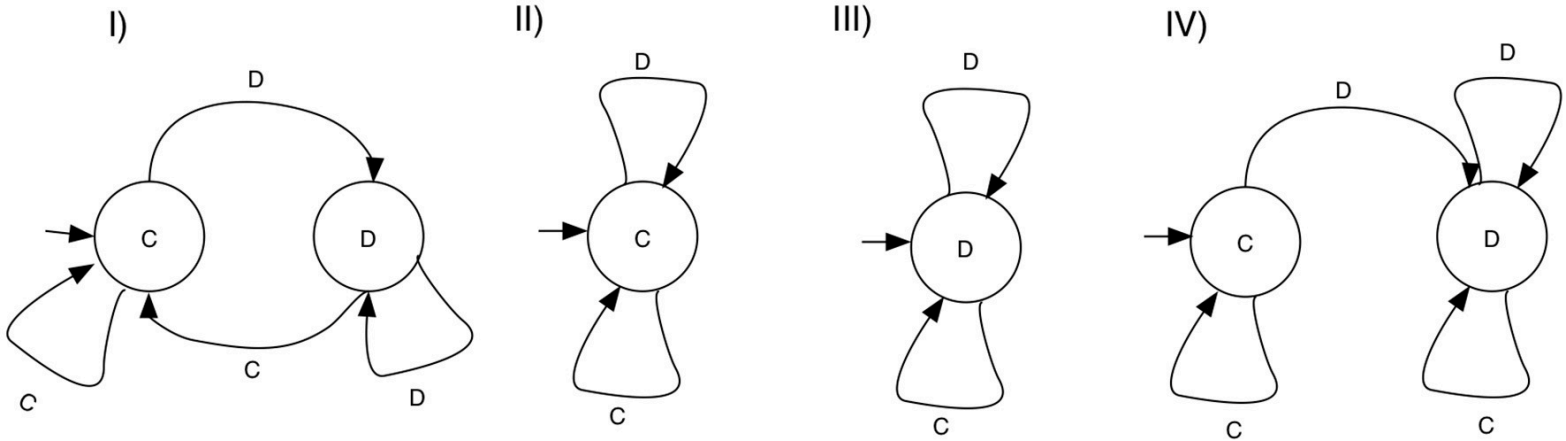
Example FSA strategies for the Repeated prisoner's dilemma. States are associated with game actions—*C* for cooperate and *D* for defect. Each state has transitions on defection and cooperation, and each machine designates an initial state. **(I)** TFT, cooperates on the first round, and repeats the move of the opponent thereafter. **(II)** ALLC, cooperates on any move. **(III)** ALLD, defects on any move, **(IV)** GRIM, cooperates as long as the opponent cooperates, and switches to defection forever if the opponent defected.

A large number of studies restrict their strategy set to those strategies that can be implemented with a finite state automaton (FSA). Here, every strategy is determined by a a set of states, together with transition rules, determining how an action from the history of the game leads to a different state of the machine. Each state is associated with an action, so termination will also determine which action is chosen in the game for any given history. A special state is designated the first action. Figure 2 shows examples of FSA's and the strategies they encode, including TFT, ALLC, ALLD, and GRIM.

Some papers study the computational complexity of implementing equilibria with FSA strategies (Gilboa, 1988; Ben-Porath, 1990), or even representations that are more powerful, such as Turing machines (Nachbar and Zame, 1996). This research shows that restricting implementations may change the game outcomes. For example, if we consider a repeated prisoners dilemma with a fixed, finite horizon, we have seen that without restrictions on the strategy set, ALLD is the only Nash equilibrium. If the number of FSA states is limited, however, it may not be possible to build a FSA that knows when it has reached the last round. In that case such a backward induction argument for ALLD being the only equilibrium in games with a finite horizon does no longer work (Shoham and Leyton-Brown, 2008), and other strategies may also be equilibria. It also shows that implementing equilibria may be computationally difficult, and therefore too demanding on boundedly rational agents (Papadimitriou, 1992).

A different line of research uses simulations to study the dynamics of a population learning to play the game. This typically simulates an evolutionary process in which strategies compete and reproduce, largely taking inspiration from evolutionary algorithms (Fogel, 2006). A solution is a set of strategies that can play the game well. The simulated selection and mutation process then gives rises to “good” solutions after a determined number of generations.

In the case of the repeated prisoner's dilemma, individuals are characterized by their strategies for the game, which are selected on the basis of the payoff they obtain, playing against other strategies in the population. Because strategies are evaluated against other strategies evolving in the population, and not against an unchanging set of opponents, this is often called “co-evolution” in evolutionary computation (Rosin and Belew, 1997).

This line of work starts with Axelrod himself (Axelrod, 1987b). Axelrod designed a simulation of strategies competing against each other. Each strategy was limited to a memory of size 3, taking into account only the last 3 moves in the history of the game. Because a single game has 4 possible outcomes (*R, S, T, P*), the strategies need to consider 4^3^ possible histories. The initial stages require a move for 6 more histories of size 2 or less (*C, D, CC, CD, DC, DD*). Thus, each strategy can be represented as a string of bits of size 70, assigning either *C* or *D* to each of the possible 70 histories. This yields 2^70^ possible strategies.

Axelrod (1987b) ran simulations where these strategies competed against each other in a process of selection and mutation. Selection uses a standard roulette-wheel procedure (Fogel, 2006), which is known in biology as Wright-Fisher process (Ewens, 2004). Crucially, he considered populations of size 20, and ran the simulations for 50–150 generations. Two main findings are reported. On the one hand, there is prevalence of “TFT-like” strategies that reciprocate against opponents, while also being cooperative on the initial moves, and forgiving if a defecting player switches back to cooperation. On the other hand, cycles appear where cooperation collapses to defection, and defection is taken over by reciprocal strategies. This is in spite of the small number of generations considered. Similar results involving cycles where found by Lindgren (1991) for memory size up to 5, including an environment where players occasionally make mistakes. For these cycles to be prevalent, it is important for the simulation run for large number of generations; e.g., up to 90, 000 in the case of Lindgren (1991).

The space of 2^70^ strategies in Axelrod's study is considerably large, but severely limits the memory of the strategies. Fogel (1993) is the first study that encodes strategies as Finite State Automata (see Figure 2), albeit limiting the size of strategies to a maximum of 8. Interestingly, some of the strategies in Axelrod (1987b) cannot be represented with this set of strategies, but dependency on histories larger than 3 is possible. Fogel (1993) also increases the population size to 100 individuals, as well as the number of generations to 200 in most simulations, with a few going up to 1, 000 generations. These experiments report the emergence of cooperation, from an initial population of defectors, but the number of generations ran is not sufficient to check if cooperation also collapses. The strategies that evolve are highly reciprocal, like TFT. An interesting outcome of this study is that there is a large range of behavioral diversity that can lead to mutual cooperation. A similar result is found in Miller (1996).

Cycles of cooperation and defection are prevalent in the literature across different approaches. These cycles involve mutual cooperation collapsing to defection and back to cooperation. In evolutionary simulations, the collapse of cooperation is often conceived as a failure of agents to learn how to cooperate (Darwen and Yao, 1995). This issue has been addressed using different computational techniques, that attempt to give reciprocal strategies the capacity to be robust and resilient to cycles. Examples include Bayesian learning (Anh et al., 2011), swarm optimization (Franken and Engelbrecht, 2005), reinforcement learning (Harper et al., 2017), amongst others (Kendall et al., 2007). We will argue that the collapse of cooperation is inherent to evolutionary learning and independent of how strategies are represented.

## 3. Game theory and simulations

Here we outline the relevant results from game theory that should be captured by a reasonable evolutionary simulation, as well as the requirements from an evolutionary simulation that is in line with the theory.

### 3.1. Rich strategy set

A repeated prisoner's dilemma is given by the game parameters *R, S, T* and *P*, as well as the continuation probability δ. We require *T*>*R*>*P*>*S*, for the stage game to be a prisoner's dilemma. This game has an action space *A* = {*C, D*}, where *C* stands for cooperation and *D* stands for defection.

With a few exceptions, most game theoretical results, presented in section 2.2, assume a complete strategy space. In order to define a (pure) strategy in this space, we first define histories of play. A history at time *t* is a list of the actions played up to and including time *t*−1. We use an empty pair of brackets to denote the empty history. The action played by player *i* at time *t* is denoted *a*_*t, i*_. So these histories are:

$$\begin{array}{lll} h_{1} & = & \left(\right)\\ h_{t} & = & \left(\left(a_{1,1},a_{1,2}\right),\ldots ,\left(a_{t-1,1},a_{t-1,2}\right)\right)\\ t & = & 2,3,\ldots \end{array}$$

This allows us to define the set of all possible histories as

$$\begin{array}{l} H=\overset{\infty }{\underset{t=1}{\cup^{\text{​}}}}H_{t} \end{array}$$

where *H*_*t*_ is the set of all possible histories at time *t*, defined as *H*_1_ = {*h*_1_}, $H_{t}=\prod_{i=1}^{t-1}\left(A\times A\right)$, for *t* = 2, 3, … . A strategy is any function *S*:*H*→*A*. This definition is as general as it gets in terms of deterministic strategies.

Not all strategies will be representable in a computer; any non-computable function (Hopcroft et al., 2001), for example, cannot be simulated, but is nonetheless included in the general set defined above.

Elsewhere, we have shown that FSA strategies are dense in the complete set of deterministic strategies. If we use a natural and appropriate metric, a deterministic strategy can be approximated arbitrarily closely by an FSA (Garćıa and van Veelen, 2016). Because of this, our simulation program will primarily use FSA's as a way to represent strategies in a computer. Importantly, the simulations described below do not assume any restrictions on the number of states in a machine, thereby providing a space as rich as possible for the evolutionary simulations. Mutations are designed so that the whole space can be explored with a positive probability. The richness of the space also implies that the number of rounds in the game should be uncertain, in order to avoid ALLD being the only equilibrium in the game.

Results will also hold for finite mixtures of FSA's (Garćıa and van Veelen, 2016). These finite mixtures are equivalent to single mixed strategies that mix (assign probabilities) in a finite number of histories, provided that there is no population structure. With population structure, equilibria in which individuals mix, and equilibria that are mixtures of pure strategies would no longer be equivalent, even if the game is not repeated (Grafen, 1979).

### 3.2. Nash equilibria are prevalent

From the folk theorem discussed above, we know that Nash equilibria are plentiful in the repeated prisoner's dilemma when δ is large enough (Fudenberg and Maskin, 1986). This should be reflected in evolutionary simulations. Being Nash does not preclude indirect invasions, but it is reasonable to expect that after an indirect invasion, there will be a (possibly short) sequence of invasions until a new Nash equilibrium is found, provided selection is doing its job (Samuelson, 2002).

To verify that this is the case, section 5.2 provides an algorithm that can check if an FSA is a Nash equilibrium for a given game. We expect that strategies that are selected for will be Nash, even if they are eventually toppled by indirect invasions (see below).

### 3.3. Cycles: no RAII, but plenty of NSS

One of the main predictions from the theory is that cycles should be prevalent. There will be plenty of neutral mutants, and because no strategy is RAII, some of those neutral mutants will open the door for other mutants with increased or decreased amounts of cooperation (Garćıa and van Veelen, 2016).

The expected, normalized payoff of strategy *S* meeting strategy *T* is:

(1) $$\begin{array}{c} \Pi (S,T)=(1-\delta )\overset{t=1}{\underset{\infty }{\sum^{\text{​}}}}\delta^{t-1}\pi (a_{t}^{S,T} \end{array})$$

Here $\pi a_{t}^{S,T}$ is the one-round payoff at time *t*, as a result of playing the actions that the unfolding of strategies *T* and *S* imply at round *t*. The factor 1−*delta* before the sum normalizes the payoffs, which allows us to compare how much cooperation there is across different δ values. With the normalization, the payoff of ALLD against ALLD is *P*, independent of δ, and the payoff of ALLC against ALLC is *R*, again, independent of δ. The normalized expected payoffs of any combination of strategies will always fall between *R* and *P*, regardless of δ.

**Figure 3:**
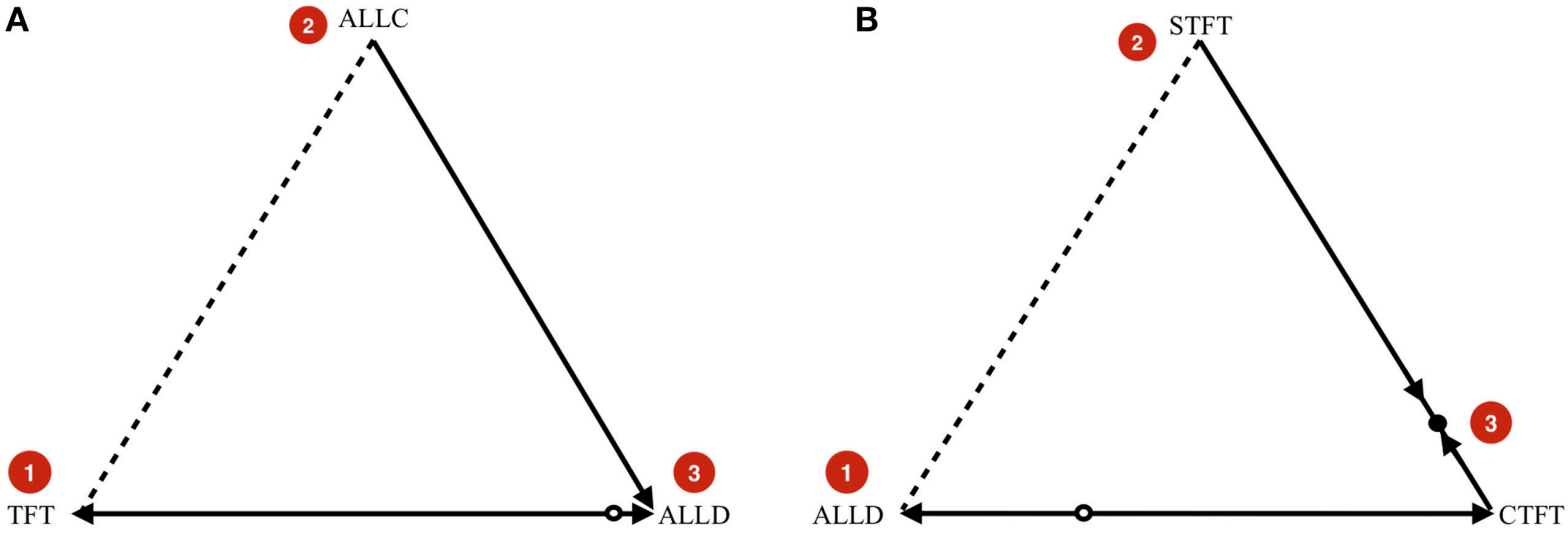
**(A)** Example indirect invasion from cooperation to defection. **(B)** Example indirect invasion from defection to cooperation. In both cases, direct invasions are impossible, but indirect invasions are possible. These examples are computed for $\delta =\frac{3}{4}$.

In the simulations, the population is large, but finite. With an infinitely large population, dynamics would be deterministic, and the theoretical benchmark for the dynamics without mutations therefore is the replicator equation (Taylor and Jonker, 1978). To describe the typical dynamical behavior we expect, we can look at the replicator dynamics on simplices that represent population states with 3 or fewer different types of strategies present (see Figure 3) and combine it with an insight from finite, stochastic population dynamics.

In Figure 3A, we start in a cooperative state where everyone is playing TFT. The strategy ALLC is a neutral mutant. Because the payoff of ALLC and TFT against TFT and against ALLC are all the same, neutral drift can make ALLC get a considerable share in the population. If it does, it opens the door for ALLD, which the dynamics predict will take over if present. The key insight is that while ALLD cannot invade TFT directly, it can invade indirectly (van Veelen, 2012), in this case via neutral mutant ALLC.

Likewise, Figure 3B shows an indirect invasion from defection to cooperation. We start in a defecting state where everyone is playing ALLD. This can be neutrally invaded by Suspicious Tit-for-Tat (STFT), a strategy that defects in the first round, and copies its opponent afterwards. A strategy that initiates cooperation can invade once STFT is sufficiently abundant in the population. Cooperate-Tit-for-Tat (CTFT) is one such strategy. It always cooperates twice at the start, and subsequently copies the opponent's action from the previous round.

The main result in Garćıa and van Veelen (2016) is that these indirect invasions should be prevalent. In an evolutionary process we should observe, over a long period of time, a succession of Nash equilibria that are toppled via indirect invasions, taking the population from defection to cooperation and back.

### 3.4. Finite but large populations

The replicator dynamics assumes an infinite population (Taylor and Jonker, 1978). The value of the relevant game theoretic solution concepts, like ESS, NSS, and RAII lies in their link to the replicator dynamics; a strategy that is ESS is guaranteed to be asymptotically stable in the replicator dynamics (Taylor and Jonker, 1978; Weibull, 1995), an NSS is Lyapounov stable (Maynard Smith, 1982; Weibull, 1995), and a strategy that is RAII is an element of an ES-set (van Veelen, 2012) that is, as a whole, asymptotically stable (Thomas, 1985; Weibull, 1995). In finite populations, random drift will make the population move within an ES-set. With the repeated prisoner's dilemma, no strategy is RAII, and therefore the simulations should consider a population that on the one hand is large enough, so that selection is strong and not too noisy, if one strategy has a selective advantage, but on the other hand is finite, so that drift can give neutral mutants a chance to open the door for other strategies.

## 4. Evolutionary simulations

Here we formulate an evolutionary simulation that complies with the conditions described above. We will use a Wright Fisher process (Imhof and Nowak, 2006), which is akin to a standard genetic algorithm, used previously in evolutionary simulations of the repeated prisoner's dilemma (Fogel, 2006).

A generation consists of *N* individuals, and is represented by a list of *N* strategies. We will assume that these strategies are finite state automata. As explained in section 3, this set is dense in the full set of deterministic strategies, provided the length of the strategies is unconstrained.

Every generation all individuals are randomly matched in pairs to play a repeated prisoners dilemma. The number of rounds in each interaction is a random variable; it follows a geometric distribution with parameter δ. The number of rounds played therefore will typically vary from pair to pair. For a pair of strategies, the expected value of the sum of the payoffs over the different rounds is given by Equation (1). Besides the noise in the number of rounds, the matching process also introduces randomness.

In the selection step, all individuals in the new generation are drawn, one by one, and independently, from a distribution where the probability of being the offspring of individual *j* from the old generation is proportional to the payoff of that individual *j*. This is known in evolutionary computation as roulette-wheel selection.

After the new generation has been drawn, any individual mutates with a small probability. This completes the cycle for a generation. The cycle is repeated a large number of times.

In the remainder of this section, we will go over the ingredients of the simulations themselves in some detail. In section 5.1, we will discuss the way the data output should be processed and analyzed.

### 4.1. Finite state automata and mutations

A FSA is a list of states. For every state it describes what the automaton plays when in that state—which is either cooperate (*C*) or defect (*D*)—to which state it goes if the opponent plays cooperate, and to which state it goes if the opponent plays defect. This makes it a proper strategy for the repeated prisoners dilemma; it returns an output for every finite string of actions that its opponent could possibly play.

The first generation is typically taken to be a population where every individual plays ALLD. There are different, but equivalent FSA, that all instantiate the strategy ALLD; every FSA for which the output in all states is *D*. When we initialize, we take the smallest, 1-state version: [*D*, 0, 0].

Every individual has a small probability to mutate. If it mutates, then one of four things happens. Either (1) a state is added, (2) the output when in a state is changed, (3) a transition is changed, or (4) a state is deleted. We chose the probability that a state is added and the probability that one is deleted such that the size of the automata does not keep growing indefinitely over a simulation run, but more or less stabilizes around a (possibly large) automaton size.

The phenomenon in which the size of the genotype tends to grow without substantially changing the phenotypic expression is known as bloat, and is common in this kind of evolutionary simulations (Poli et al., 2008). Importantly, the choice to nudge the simulations into avoiding overly long automata is driven only by the need to produce simulations that run in a reasonable time. Computing payoffs for large automata is more costly, and ever expanding automata over long runs make the simulations slow down ever more. The cognitive interpretations of different exploration or mutation schemes represent an open problem that will be discussed in section 6. As it will be highlighted later, the analysis in section 5.1 uses the minimal representation of automata.

**Figure 4:**
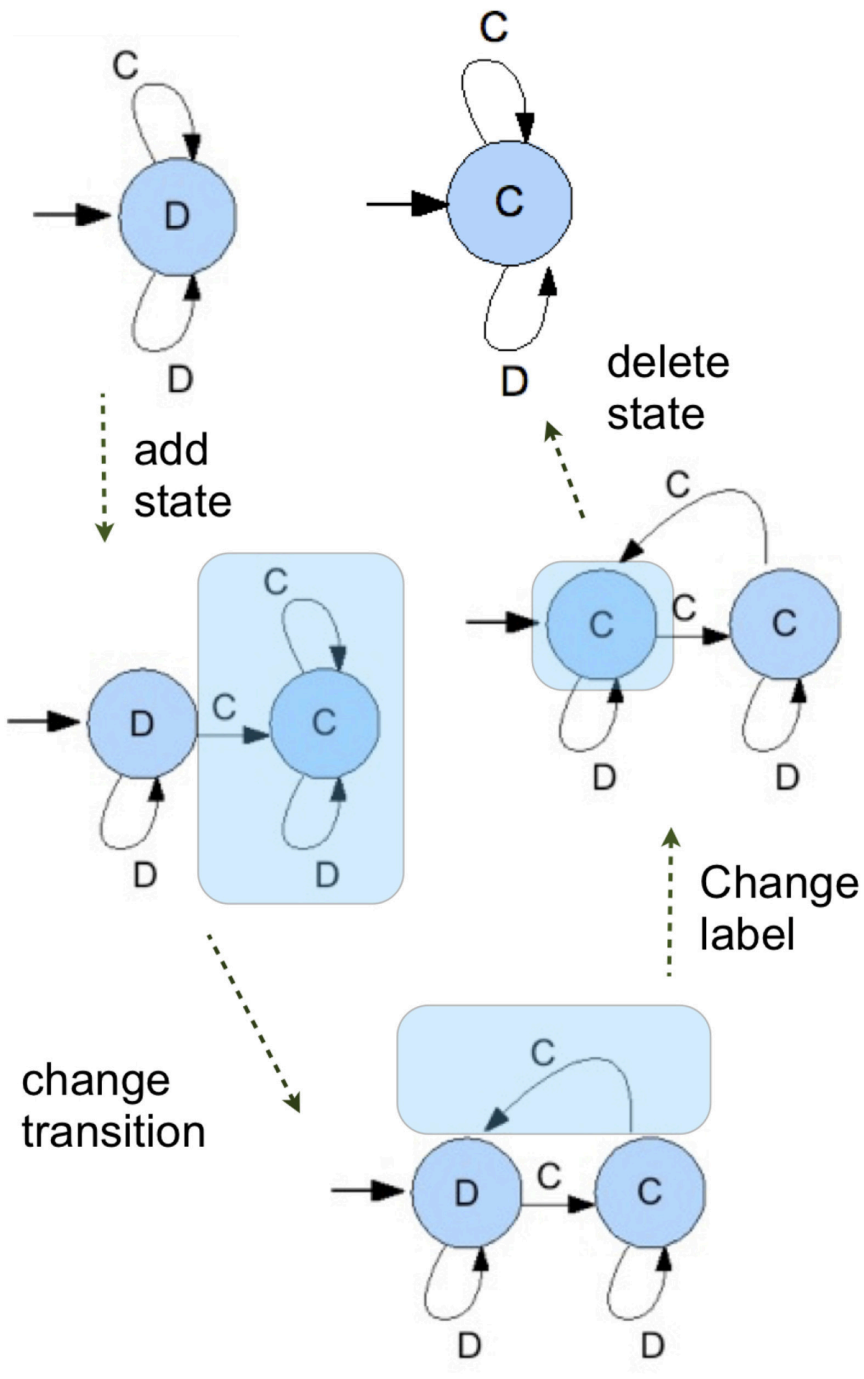
A series of mutation operations show a possible path in which and ALLD strategy is transformed into an ALLC strategy, showing all possible mutations.

The scheme of mutations is illustrated in Figure 4. The advantage of this mutation scheme in combination with the setup where the population is a list of actual individuals with possibly different strategies is that this allows the simulations to explore the richness of the strategy set. With those four ways to mutates, there is a sequence of mutations between any two FSA's. That means that every FSA has a positive probability of mutating into the population in a finite number of mutation steps. It should be said, however, that the probabilities with which any given mutant enters the population depends on the current population, since all mutations have to work with what is there—as they do in nature.

We ignore crossover, whereby new mutants take material from two existing strategies (Fogel, 2006). Allowing crossover as one of the possibe ways to mutate would change the relative likelihood of different mutations, but since stepping stone paths out are already found without crossover, there is no reason to expect that the dynamics will be fundamentally different with crossover.

### 4.2. The game

A simulation allows the stage game to be any 2 × 2 matrix game, but for this paper we restrict ourselves to the prisoners dilemma. For continuation probabilities δ < 1 the number of repetitions between any pair of two agents is a random variable. It is a geometric distribution with mean 1/δ; the probability that the interaction lasts exactly *i* rounds is δ^*i*−1^(1−δ). For δ = 1 we compute the “limit of means” payoffs. For any combination of two FSA's, there is a moment in time where play between them starts repeating itself; if one FSA has *n* states, and the other has *m*, then there are only *n*×*m* combinations of states that they could be in jointly, so at some point they will start cycling. We therefore take the average payoff over the cycle.

### 4.3. The selection step

For the update step we use the Wright-Fisher process—equivalent to roulette wheel selection in evolutionary algorithms. All individuals in the new generation are drawn, one by one, and independently, with a probability proportional to payoff. More specifically, if π_*i*_ is the payoff earned by individual *i* in the previous generation, at every draw, the probability that individual *i* will be chosen for the new generation is $\pi_{i}/\overset{N}{\underset{j=1}{\sum }}\pi_{j}$.

The best known alternative is the Moran process, where only one individual reproduces in every cycle. While this process is widely used in exact calculations, it is prohibitively inefficient for Monte Carlo simulations. In the Moran process, the matching as well as the unfolding of the game are to be repeated all over again for a single replacement in the population, while in the Wright-Fisher process a whole generation is replaced based on the matching and the payoffs. Given that the only difference between the processes is the speed, we report results using the Wright-Fisher process. This process is efficient for simulations and also closer to the standard literature in evolutionary computation.

**Figure 5:**
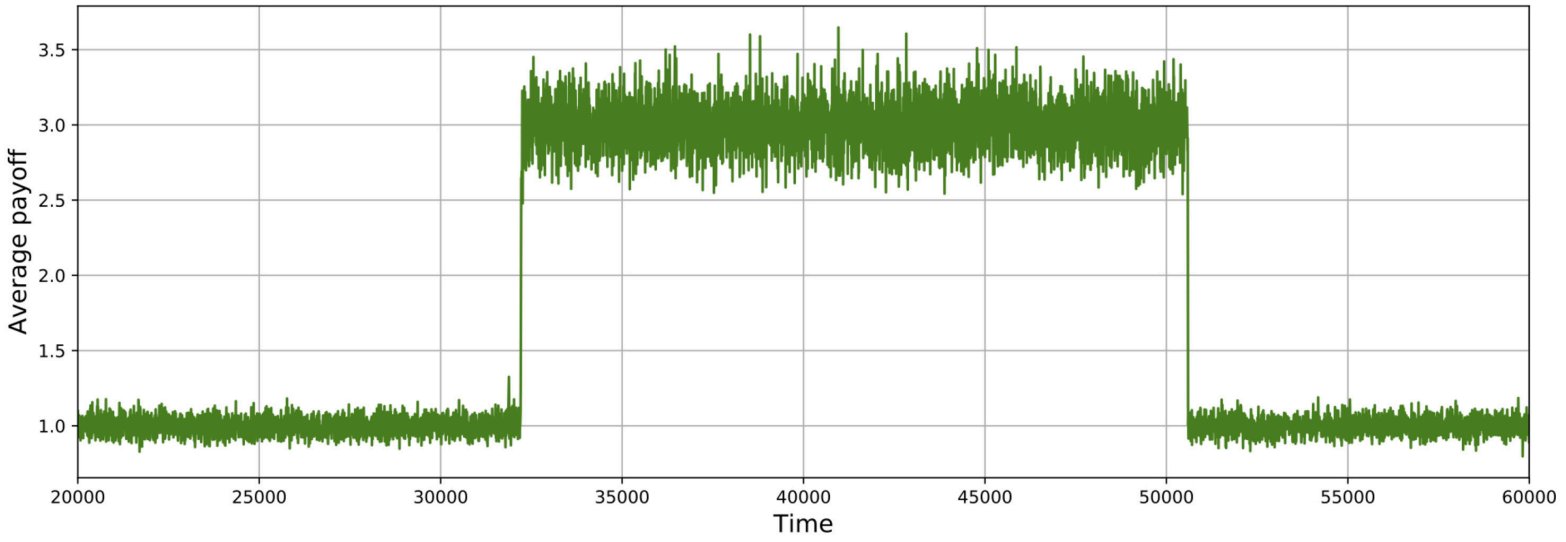
Average payoff through time, where higher payoffs imply more cooperation. A slice of a typical simulation shows cycles of defection and cooperation. In this case, the population size is 512, δ = 0.75, mutation probability is set to 10^−4^, and the prisoner's dilemma is given by *R* = 3, *S* = 0, *T* = 4, and *P* = 1.

**Figure 6:**
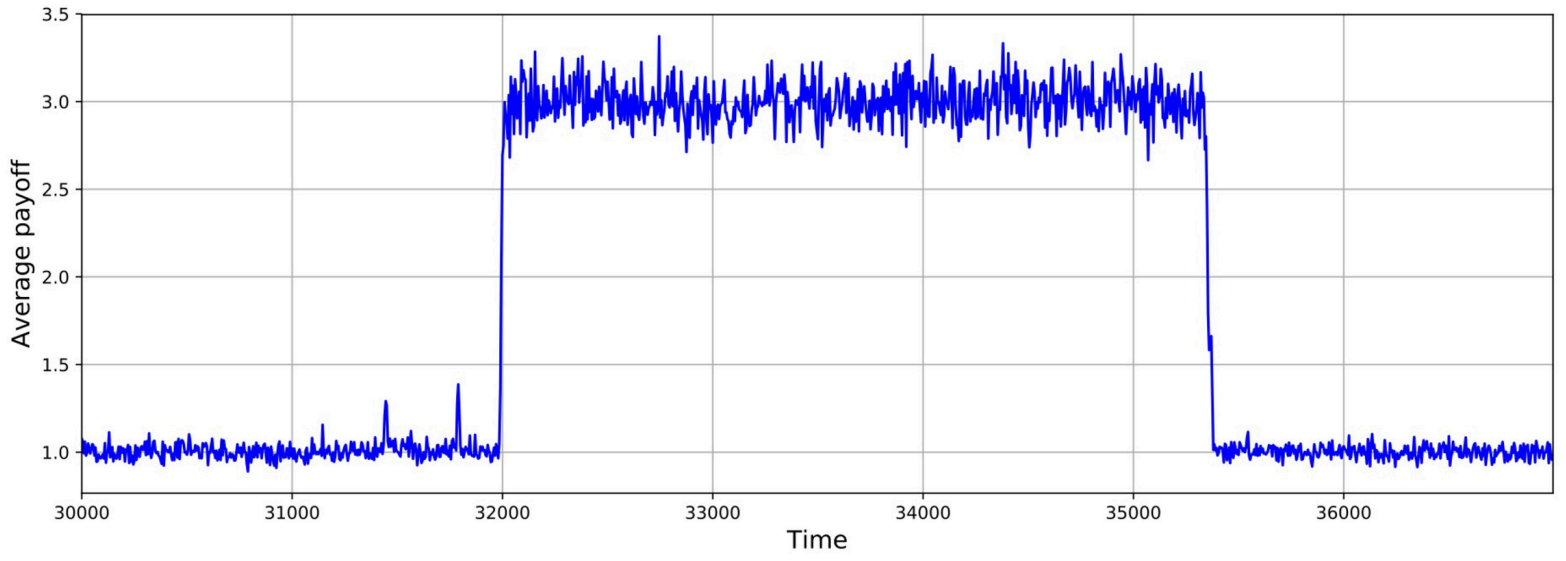
Average payoff through time, where higher payoffs imply more cooperation. A slice of a typical simulation shows cycles of defection and cooperation. In this case, the population size is 1, 024, δ = 0.75, mutation probability is set to 10^−4^, and the prisoner's dilemma is given by *R* = 3, *S* = 0, *T* = 4, and *P* = 1.

As shown in Figures 5, 6, these simulations show cycles of defection and cooperation. As expected, less demographic noise in larger populations leads to smoother dynamics, because the noise is averaged out.

All in all, the recipe we present for simulations incorporates elements introduced elsewhere in the simulation literature (Axelrod, 1987a; Lindgren, 1991; Miller, 1996; Fogel, 2006). They crucially differ in allowing for an unbounded space, considering a game with discounting, and running the simulations long enough to observe cycles. Next, we will show how to verify that these simulations align with theory.

## 5. Analysing simulation data

Having formulated a suitable evolutionary simulation, verifying that game theoretic predictions are aligned with simulation outcomes requires us to inspect the data in a way that highlights indirect invasions as the the main drivers of the dynamics. Here we describe how such analysis can take place.

### 5.1. Capturing transitions and indirect invasions

#### Step 1: detecting equilibria

The aim of the simulations is to find out if the possibility of indirect invasions indeed shapes evolutionary dynamics in repeated games. Before being able to say if an equilibrium was left through an indirect invasion, it is important to first be able to say if it was left at all. While transitions are made possible by a mutation process that constantly produces new strategies, that very same production of new strategies also creates noise in the population. This implies that if we think for instance of a pure equilibrium, we should not only classify a population as being at that equilibrium if the population consists of that one strategy only and nothing else. Given the frequent introduction of mutants, most of which enter only to be eliminated from the population before ever having attained a considerable share, we should also classify nearby population states as being at that equilibrium, and create a bandwidth which allows us to disregard this noise.

If the population at time *t* consists of strategy *A* only, and at time *t*+100 of strategy *B* only, then it is fair to say that at least one transition has occurred. If on the other hand the population at time *t*, and at time *t*+100, and at all times in between, consists of between 90 and 100% strategy *A*, plus a remainder that is composed of an ever changing set of other strategies, then it seems reasonable to assume that a transition has not occurred, and that the little differences only reflect the regular influx and extinction of new mutations.

We therefore begin the classification of a population state by ranking the composing strategies from frequent to infrequent. Then we look at the minimum number of strategies that is needed to capture at least a fixed percentage of the population (we choose 90% for the threshold). A population state is then characterized by how many strategies are needed to reach this percentage (1 strategy, 2 strategies, 3 strategies) and which those strategies are, ordered from most popular to least popular. In case more than 3 strategies were needed, this was classified under “other interior states”. With a threshold of 90%, a population that, for example, consists of 65% strategy *A*, 30% strategy *B*, and 5% strategy *C* is classified as a mixture of 2 strategies; *A* (most popular) and *B* (second most popular). The classification thereby never ignores more than 10% of the population. With small but positive mutation rates and population sizes in the simulations, a population where the three most popular strategies made up less than 90% of the population is a rare exception.

**Figure 7:**
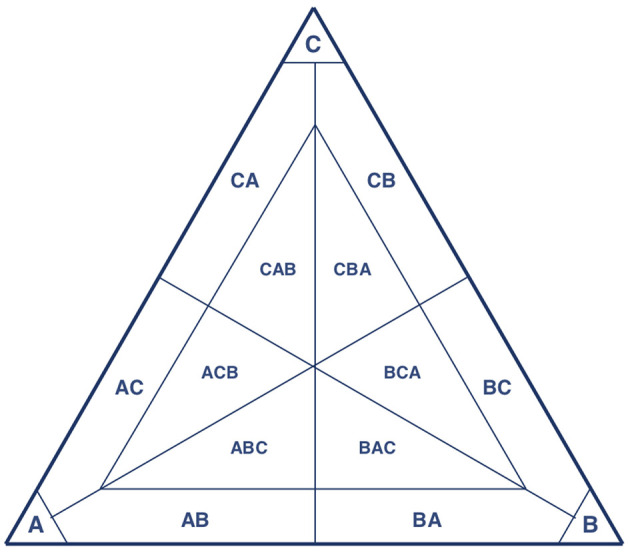
With only three strategies present, this depicts the classification of population states with a threshold of 90%.

This classification allows us, at least to some extent, to pick up three types of (possible) equilibria; pure ones, mixed ones with two strategies, and mixed ones with three strategies. If the population is at a pure equilibrium, we expect that it finds itself in a corner pocket (see Figure 7), and that most of the mutants do not take the population outside this corner pocket, provided mutation rates are small enough (Wu et al., 2012; Vasconcelos et al., 2017). If a population is a mixed equilibrium with two strategies, it should find itself somewhere in between two vertices. The construction of the pocket implies that we cannot capture mixed equilibria where one of the pure strategies would make up less than 10% of the population in equilibrium.

With this way to classify population states, we can follow the population as it travels from region to region. At any such transition, we can check if this transition can be associated with a neutral mutant entering or exiting the population, or with advantageous or disadvantageous mutants entering or exiting the population. This gives us the possibility to characterize a sequence of transitions as an indirect invasion or an invasion of a different kind. If strategy *A* is a pure equilibrium strategy, and *B* is a neutral mutant of *A*, and *C* has a strict advantage against *B*, then—with obvious abbreviations—going from the region *A* to *AB* to *BA* to *B* to *BC* will be classified as an indirect invasion^1^. If *A* is a pure equilibrium, and *D* is a mutant with a selective disadvantage, then going from region *A* to region *AD* is classified as a different invasion.

This immediately points out the trade-off that we face for the choice of a threshold. If we choose a threshold that is larger than $\frac{n-1}{n}$, where *n* is the population size, then any mutant entering the population will take it outside the corner pocket. All mutants will therefore be recorded as transitions, and all disadvantageous mutants will be recorded as “different invasions”, even though they might be extinct the next generation already. This high threshold thereby leaves no room at all to observe what we are interested in, which is the difference in how selection acts on different (sequences of) mutants. On the other hand, if we choose the threshold as low as 50%, then we leave no room to observe dynamics near mixed equilibria at all, as described above. So a lower threshold means more room to observe selection at work, but also more mixed equilibria that will go unnoticed, because they end up in corner pockets.

From the simulations we know that mixed equilibria with two strategies outside the corner pockets, where both strategies account for more than 10% of the population, are typically left because one of the composing pure strategies fixates. This is caused by noise, and such paths out of a mixed equilibrium will get less likely in larger populations, since it involves the population moving against the direction of selection. Therefore, the larger the population, the longer the mixtures tend to stick around. It seems natural to expect that mixed equilibria where one strategy accounts for less than 10% of the population are left through fixation even more easily. Note also that subdivisions of the simplex, as pictured in Figure 7, are unavoidable if we want to analyse simulation output; we need to be able to say when an equilibrium is left.

#### Step 2: counting transitions

Once we have changed the raw data into a sequence of “regions,” we need to count transitions out of equilibria. As starting points of paths out of equilibrium, we only chose equilibria that were not themselves reached by a neutral invasion. The reason is that it is very well possible that an equilibrium is invaded by a neutral mutant that still is an equilibrium itself. In fact, equilibria typically are followed by a sequence of neutral mutants that have the same self-play, which, for as long as they are equilibria, is the equilibrium path. This implies that if we find a sequence of neutral mutants that themselves are equilibria, which is followed, first, by a neutral invasion to a state that is not an equilibrium, which in turn is followed by an advantageous mutant, then it is reasonable to count the whole sequence as one single indirect invasion. It is for sure an indirect invasion starting from the last equilibrium, and also one when we start from the first one, and from all equilibria in between, but counting it as just one single indirect invasion is more than reasonable. (All other sequences out of equilibrium—zero or more neutral mutants followed by the entry of disadvantageous mutant—are qualified as “other invasions”).

For a more formal version, we can, for a strategy *P* (which is possibly a mixture over pure strategies) define the set of (evolutionary) worse, equal, and better performers against *P*^2^.

$$\begin{array}{l} S_{W}\left(P\right)=\{Q\;|\;U\left(Q,P\right)<U\left(P,P\right)\text{ or }(U\left(Q,P\right)\\ \;=U\left(P,P\right)\text{ and }U\left(Q,Q\right)<U\left(P,Q\right))\}\\ S_{E}\left(P\right)=\left\{Q\;|\;U\left(Q,P\right)=U\left(P,P\right)\text{ and }U\left(Q,Q\right)=U\left(P,Q\right)\right\}\\ S_{B}\left(P\right)=\{Q\;|\;U\left(Q,P\right)>U\left(P,P\right)\text{ or }(U\left(Q,P\right)\\ \;=U\left(P,P\right)\text{ and }U\left(Q,Q\right)>U\left(P,Q\right))\} \end{array}$$

An observed sequence of strategies *O, P, Q*^1^, …, *Q*^*n*^ is counted as one indirect invasion if *P* is an equilibrium, if *P*∉*S*_*E*_(*O*)—that is, *P* is not reached by a neutral invasion itself—if $Q^{1}\in S_{E}\left(P\right)$, $Q^{i}\in S_{E}\left(Q^{i-1}\right)$ for 2 ≤ *i* ≤ *n*−1—that is, *Q*^1^ to *Q*^*n*−1^ is a sequence of neutral mutants—and if $Q^{n}\in S_{B}\left(Q^{n-1}\right)$—that is, *Q*^*n*^ is an advantageous mutant. Some of those *Q*^*i*^ can themselves be equilibria too. Similarly, an observed sequence of strategies *O, P, Q*^1^, …, *Q*^*n*^ is counted as one single other path out of equilibrium if *P* is an equilibrium, *P*∉*S*_*E*_(*O*), $Q^{1}\in S_{E}\left(P\right)$, $Q^{i}\in S_{E}\left(Q^{i-1}\right)$ for 2 ≤ *i* ≤ *n*−1, and $Q^{n}\in S_{W}\left(Q^{n-1}\right)$. Again, some *Q*^*i*^ for 2 ≤ *i* ≤ *n*−1 can be equilibria themselves too. Obviously, a sequence of strategies *O, P, Q*, with *P*∉*S*_*E*_(*O*) and *Q*∈*S*_*W*_(*P*) also counts as one path out of equilibrium that is not an indirect invasion.

### 5.2. Verifying nash equilibria

In order to be able to determine if a finite automaton—and hence a pure strategy—is a Nash equilibrium, we present an algorithm called the best responder. This algorithm finds the payoff of the best response against strategy *S*, as well as the best response itself. If the payoff of *S* against itself equals this payoff, then *S* is a Nash equilibrium. This is a useful device, since the infinity of the strategy space does not allow us to simply compare the payoff of *S* against itself to the payoff of all other strategies against *S* one after the other.

Suppose strategy *S* is an automaton with *N*_*S*_ states. Any state *i* is characterized by an action played by *S* when it finds itself in this state—λ_*S*_:{1, …, *N*_*S*_} → {*C, D*}—and a list of transitions as a function of the action played by the opponent of *S*—μ_*S*_:{1, …, *N*_*S*_} × {*C, D*} → {1, …, *N*_*S*_}.

The value to the opponent of strategy *S* of *S* being in state *i* is denoted by $V_{S}^{*}\left(i\right),i=1,\ldots ,N_{S}$. We aim to find these values as a solution to the following system:

$$\begin{array}{l} V_{S}\left(i\right)=\underset{a\in \left\{C,D\right\}}{\max}\left\{\pi_{1}\left(a,\lambda_{S}\left(i\right)\right)+\delta V_{S}\left(\mu_{S}\left(i,a\right)\right)\right\}i=1,\ldots ,N_{S} \end{array}$$

Let $V_{S}^{*}\left(i\right),i=1,\ldots ,N_{S}$ be the solution to this system. The discounted value in the initial state, $\left(1-\delta \right)V_{S}^{*}\left(1\right)$, is the maximal discounted payoff that can be earned against *S*, and

$$\begin{array}{l} a_{i}^{*}=\arg\underset{a\in A}{\max}\left\{\pi_{1}\left(a,\lambda_{S}\left(i\right)\right)+\delta V_{S}^{*}\left(\mu_{S}\left(i,a\right)\right)\right\} \end{array}$$

gives the optimal action when *S* is in state *i*.

The best responder does the following iteration.

Initialization step:

$$\begin{array}{l} V_{S,1}\left(i\right)=0,\;i=1,\ldots ,N_{S} \end{array}$$

Iteration step:

$$\begin{array}{l} V_{S,n+1}\left(i\right)=\underset{a\in A}{\max}\left\{\pi_{1}\left(a,\lambda_{S}\left(i\right)\right)+\delta V_{S,n}\left(\mu_{S}\left(i,a\right)\right)\right\}\;i=1,\ldots ,N_{S} \end{array}$$

where *V*_*S, n*_(*i*) is the value to the opponent of strategy *S* of *S* being in state *i*, at step *n* in the iteration.

It is quite straightforward that this iteration converges, as is shown in the following simple lemma. We will assume that the initialization makes sure that we begin with values for all states that are below the solution of the system (whenever this procedure is invoked, we make sure that is in fact the case) but that is not actually necessary for convergence.

**Lemma 1**. If $V_{S,1}\left(i\right)\leq V_{S}^{*}\left(i\right)$ for all *i* and if δ∈[0, 1) then the above iteration converges to $V_{S}^{*}\left(i\right),i=1,\ldots ,N_{S}$.

*Proof*. First, if $V_{S,n}\left(i\right)\leq V^{*}\left(i\right)$ for all *i*, then also

$$\begin{array}{l} V_{S,n+1}(i)=\underset{a\in A}{\max}\{\pi_{1}(a,\lambda_{S}(i))+\delta V_{S,n}(\mu_{S}(i,a))\}\\ \;\leq \underset{a\in A}{\max}\{\pi_{1}(a,\lambda_{S}(i))+\delta V_{S}^{*}(\mu_{S}(i,a))\}=V^{*}(i)\\ \text{ for all}i\text{.} \end{array}$$

Hence $V_{S}^{*}\left(i\right)-V_{S,n}\left(i\right)\geq 0$ for all states *i* and all iterations *n*.

By definition we also have

$$\begin{array}{l} V_{S,n+1}\left(i\right)\geq \pi_{1}\left(a_{i}^{*},\lambda_{S}\left(i\right)\right)+\delta V_{S,n}\left(\mu_{S}\left(i,a_{i}^{*}\right)\right)\text{for all}i\text{.} \end{array}$$

Therefore

$$\begin{array}{l} 0\leq V_{S}^{*}(i)-V_{S,n+1}(i)\leq \delta (V_{S}^{*}(\mu_{S}(i,a_{i}^{*}))-V_{S,n}(\mu_{S}(i,a_{i}^{*})))\\ \text{ for all}i\text{.} \end{array}$$

This implies that

$$\begin{array}{l} 0\leq \underset{i}{\max}\left(V_{S}^{*}\left(i\right)-V_{S,n+1}\left(i\right)\right)\leq \delta \max\left(\overset{*}{\underset{S}{V}}\left(i\right)-V_{S,n}\left(i\right)\right) \end{array}$$

and since 0 < δ < 1 we find that $\underset{n\to \infty }{\lim}\left(V_{S}^{*}\left(i\right)-V_{S,n}\left(i\right)\right)=0$ for all *i*.     □

The best responder gives us both the maximum payoff $\left(1-\delta \right)V_{S}^{*}\left(1\right)$ when playing against *S*, and an optimal strategy when playing against *S*, as $a_{i}^{*}$ prescribes what to play when *S* is in state *i*. For numerical reasons, we use the optimal strategy against *S* when we evaluate whether *S* is a best response to itself. For that, it is important to be able to distinguish between the payoff of *S* against itself being exactly equal to the maximum payoff when playing against *S*, or smaller. If we were to use the maximum payoff $\left(1-\delta \right)V_{S}^{*}\left(1\right)$ that results from this iteration directly, then this will have some numerical inaccuracy in it. Comparing that maximum payoff against *S* to the payoff of *S* against *S* may lead to an incorect outcome, because the latter is computed by simply evaluating a discounted stream of payoffs, which will also have some numerical inaccuracy in it, but typically a different one than the iteration. We therefore may inadvertedly find these to be different, when they really should be the same number. In order to avoid that, we use the optimal strategy against *S* that the iteration produces, first let it play against *S*, then let *S* play against itself, and compare the two payoffs. This way, they will have the *same* inaccuracies in both, because both procedures of evaluating the discounted payoffs are the same.

Note that the algorithm works with phenotypes, not with genotypes, so two different ways to encode for instance the strategy ALLD will be treated as one and the same strategy. To allow for this, we minimize the FSA representing the strategy, and compare only minimal implementations (Hopcroft et al., 2001).

**Figure 8:**
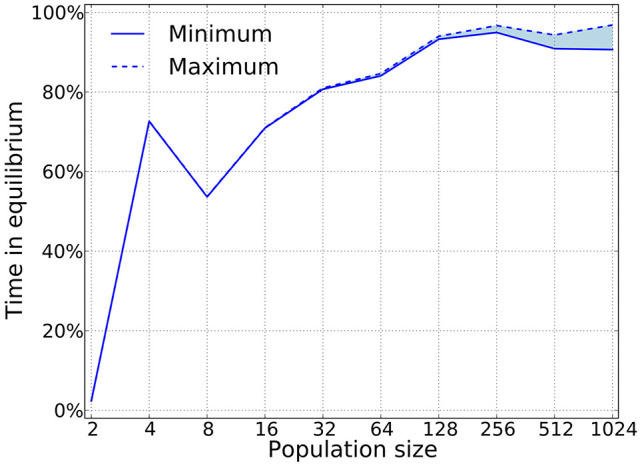
Nash equilibria are prevalent as predicted by the theory. Our algorithm only works for pure strategies, which implies that we can provide a lower bound on the time that is spent in equilibria, as mixed equilibria will naturally arise in a large strategy space. For this simulations δ = 0.75, mutation probability is set to 10^−4^, and the prisoner's dilemma is given by *R* = 3, *S* = 0, *T* = 4, and *P* = 1.

Using this algorithm we can check whether or not pure strategies that appear in the simulation are are Nash. Figure 8 shows that for large population sizes, the dynamics is composed almost exclusively of Nash equilibria. This result is expected to hold with sufficiently small mutation rates, since we want selection to dominate the process. Also, as expected, this shows that large populations are required to meet the predictions from game theory. Previous work has shown deviations between simulations and game-theoretical predictions when the population size is small Fogel et al. (1998).

The best responder only works for pure strategies, so for mixed population states, we have no automated check whether or not they are a Nash equilibrium. That implies that for the time the simulation spends in mixed states, we do not know if this is an equilibrium state or not.

Following the recipe articulated in sections 5.1 and 5.2, Garćıa and van Veelen (2016) show that indirect invasions account for more than 80% of the transitions in cycles when the population size is above 500, and up to 100% for a population size of 1,000 individuals.

Indirect invasions dominate the dynamics, taking the population from one Nash equilibria to another, with varying different levels of cooperation. This is in line with the theory that predicts no stability, regardless of how strategies are represented.

## 6. Discussion

We provide algorithms for analysing evolutionary simulations. Using this it can be shown that game theoretical concepts are in line with the cycles that are typically observed in computer simulations and approaches inspired in evolutionary computation. Paths in and out of cooperation crucially depend on the right sequence of strategies arising. The typical route involves a neutral mutant that catalyses the collapse of the resident strategy.

To verify this it is important to run the simulations long enough so that cycles can appear. Once these appear, the right algorithms can show that all cycles follow a certain path where neutral strategies are important in toppling strategies out of their prevalence. To detect these cycles, we use an algorithm that identifies transitions (section 5.1), and another one to show that for large populations the dynamics is dominated by Nash equilibria (section 5.2).

Our work also shows that simulation models are compatible with game theory analysis. Not only can game theory make it easier to focus on the right kind of data analysis of simulation results, but simulations themselves can also help us push game theory forward. Our view is that more attention should be paid to how representations and mutations may affect the odds for cooperation to arise (e.g., Garćıa and Traulsen, 2012). While cycles are unavoidable in repeated games, different mutation schemes—and strategy representations—may lead to more or less cooperation.

Our analysis shows that cycles are prevalent when using the most general space of deterministic strategies, including non-computable strategies. This implies that cognition itself may bear little impact in changing the cyclic dynamics. The collapse of cooperation is unavoidable under evolutionary learning, regardless of how smart strategies are. Research should therefore focus on understanding the process of strategy exploration and implementation.

To see this, notice that although the set of FSA strategies ignores, for example, strategies that can count (e.g., implemented using Pushdown Automata), the theory of indirect invasions also holds in that case. Therefore, under a reasonable scheme of mutations, smarter strategies will ultimately also succumb to cycles. We can expect this to hold for any kind of machine, including Turing machines, because they are all subsets of the functional strategy definition. Levels of cooperation may vary according to mutation schemes, but these are not directly concerned with cognition or with how smart the strategies *per se* are. Cycles are to be expected either way.

We have chosen to present results using the Wright Fisher process, because this turns out to be more computationally efficient when doing Monte Carlo simulations, as compared to the Moran process. In terms of the long-term outcomes, we do not expect major differences if using a process in which fitter individuals reproduce more and noise is not prevalent. This is because the game theoretical results do not depend on any specific choice of the process, relying only on selection itself. Exploring processes other than the Wright Fisher process may lead to differences in details, such as the speed of convergence. The specific effects of different implementations of the selection process are an important topic for future research.

There is no winning strategy in the repeated prisoner's dilemma, because every strategy can be overturned by the right sequence of mutants. Some exploration process may be more conducive than others to cooperation. The cognitive aspects of innovation and exploration are therefore more important in this problem than the cognitive aspects of implementing strategies.

## Author contributions

All authors listed have made a substantial, direct and intellectual contribution to the work, and approved it for publication.

### Conflict of interest statement

The authors declare that the research was conducted in the absence of any commercial or financial relationships that could be construed as a potential conflict of interest.

## Footnotes

^1^If *C* already has a selective advantage when the population is in *BA*, then also a sequence of *A* to *AB* to *BA* to *BAC* (or to *BC*) counts as an indirect invasion.

^2^This *P* is a distribution over strategies, as in Garćıa and van Veelen (2016), and is not to be confused with the payoff *P* from the prisoner's dilemma.
